# Supplementary material for: Chromothripsis during telomere crisis is independent of NHEJ, and consistent with a replicative origin
Source: Genome Res. 2019 May;29(5):737–49. doi: 10.1101/gr.240705.118 (PMC6499312; doi:10.1101/gr.240705.118)
Supplement: Supplemental Material [file supp_gr.240705.118_Supplemental_file_1.zip › contigs/annotated_contigs/DB53/contig.3.DB53_length_413_mean_cov_4.3583535109.docx]

**DB53_length_413_mean_cov_4.3583535109**

```
TACTCTTTTCACCCAGGATTATATTTTAAGACGGTCTTTGTTGCTGTCAATATCG|C|GATTTTTAGGAAGATGATGATCAATTAACAA
 >chr7:155908846-155908901 - E=1e-21                     >chr8:135432456-135432679 + E=
TGCCCAGTGGTACCTACAGGCCAAGACTTTGTGAATATCAGTGTTGCCTTTATGGTAAATGCACCCATCTTATTTTTCTTTCCAGGAAG
9e-123
TGCTCTTGACTTCTACTACTCCAGATTCCTATAATCTGAAATGAAATCAAAGAGTCCATCTCAATAGGATTCCCCTATTATCACACTTG

GCCTACTAAGAAGA|TG|CCCACCATGCCTGCCTAATTATAAATAACCAAAAAATATCTCAGGCACTGTCCAATACCACTTTACCTCAT
                 >chr8:136010671-136010803 - E=6e-68
GTATCAATGACCTAAGATGATCATTGATAATATTTCATGAGTTCAGCTTAGCATTTATTTA
```
